# Targeting Cytokinin Homeostasis in Rapid Cycling *Brassica rapa* with Plant Growth Regulators INCYDE and TD-K

**DOI:** 10.3390/plants10010039

**Published:** 2020-12-25

**Authors:** Matthew J. van Voorthuizen, Jaroslav Nisler, Jiancheng Song, Lukáš Spíchal, Paula E. Jameson

**Affiliations:** 1 School of Biological Sciences, University of Canterbury, Christchurch 8140, New Zealand; matthewmjvv@gmail.com (M.J.v.V.); jcsong88@163.com (J.S.); 2 Laboratory of Growth Regulators, Centre of the Region Haná for Biotechnological and Agricultural Research, Institute of Experimental Botany AS CR & Palacký University, CZ-783-71 Olomouc, Czech Republic; jaroslav.nisler@gmail.com; 3 Centre of the Region Haná for Biotechnological and Agricultural Research, Department of Chemical Biology and Genetics, Palacký University, CZ-783 71 Olomouc, Czech Republic; lukas.spichal@upol.cz; 4 School of Life Sciences, Yantai University, Yantai 264005, China

**Keywords:** cytokinin, INCYDE, TD-K, thidiazuron, isopentenyl transferase, IPT, cytokinin oxidase/dehydrogenase, CKX

## Abstract

Modifying the cytokinin content in plants is a means of improving plant productivity. Here, we report the development and biological activity of compound TD-K (1-(furan-2-ylmethyl)-3-(1,2,3-thiadiazol-5-yl)urea)which is related to thidiazuron. TD-K—which exhibited extremely high antisenescence activity in the wheat leaf bioassay—and INCYDE (2-chloro-6-(3-methoxyphenyl)aminopurine)—a plant growth regulator reported to inhibit cytokinin oxidase/dehydrogenase (CKX), an enzyme involved in the degradation of the plant hormone cytokinin—were selected for investigation of their effects on the model plant Rapid Cycling *Brassica rapa* (RCBr). We monitored the expression of *BrCKX* and isopentenyl transferase (*BrIPT*), which codes for the key cytokinin biosynthesis enzyme, in developing leaves following INCYDE and TD-K application. Growth room experiments revealed that INCYDE increased RCBr seed yield per plant, but only when applied multiple times and when grown in 5 mM KNO_3_. Expression in control leaves showed transient, high levels of expression of *BrCKX* and *BrIPT* at true leaf appearance. Following INCYDE application, there was a rapid and strong upregulation of *BrCKX3*, and a transient downregulation of *BrIPT1* and *BrIPT3*. Interestingly, the upregulation of *BrCKX3* persisted in a milder form throughout the course of the experiment (16 days). TD-K also upregulated *BrCKX3.* However, in contrast to INCYDE, this effect disappeared after two days. These results suggest that both compounds (CKX inhibitor and cytokinin TD-K) influenced cytokinin homeostasis in RCBr leaves, but with different mechanisms.

## 1. Introduction

Challenges posed by an increasing global population, climate change and loss of biodiversity underline the need for innovations that will lead to significant improvements in yield and productivity in agriculture [1,2]. The cytokinins are a plant hormone involved in a number of fundamental processes associated with yield. These include seed number and size [3], shoot and root development [4], branching and apical dominance [5], senescence [6,7], shoot apical meristem and flower development [8], abiotic and biotic stress response [9] and nitrogen status communication [10].

The naturally occurring cytokinins are adenine derivatives that can be categorized into those with an isoprenoid side chain and those with an aromatic one [11,12]. The isoprenoid cytokinins occur in free base, riboside and nucleotide forms. The free bases, *trans*-zeatin (*t*Z), *cis*-zeatin (*c*Z), dihydrozeatin (DHZ) and *N*^6^-isopentenyladenine (iP) which differ in their side chains, are considered the active forms detected by histidine kinase receptors [13]. *Trans*-Z-type and iP-type cytokinins are considered the major cytokinins in angiosperms [14], while roles for *cis*-zeatin-types, although ubiquitous [15], are yet to be firmly established [16]. Aromatic cytokinins include *N*^6^-benzyladenine (BA), the topolins, and the synthetic kinetin (K) [11,17]. Cytokinin-active molecules also include INCYDE (Inhibitor of Cytokinin Dehydrogenase), and synthetic phenylurea derivatives such as CPPU (1-(2-chloropyridin-4-yl)-3-phenylurea) and thidiazuron (TDZ, 1-phenyl-3-(1,2,3-thiadiazol-5-yl) urea), all of which are recognized by cytokinin receptors [18,19,20].

Cytokinin biosynthesis is catalyzed by isopentenyl transferases (IPTs and tRNA-IPTs) [14,21]. *IPT* is encoded by a small multigene family [22,23], with tissue- and developmentally specific expression patterns [24]. The first formed, inactive nucleotides are activated by LONELY GUY (LOG) [25] to the free base forms.

The degradation of cytokinin is catalyzed by cytokinin oxidase/dehydrogenases (CKXs), an enzyme that irreversibly cleaves the *N*^6^-side chains of both free-base and riboside cytokinins [26,27]. Several cytokinins are known to be resistant to cleavage by CKX including DHZ, BA and kinetin [28,29]. A small gene family encodes *CKX* [30], with gene family members (GFMs) spatio-temporally differentiated in expression [31]. The overexpression of *CKX* results in deficiencies in shoot development but enhancement of root growth [4,32].

Numerous studies suggest that *IPT*s and *CKX*s play an important role in determining seed number and also seed size [3], and the disruption of cytokinin homeostasis, therefore, provides important opportunities for yield enhancement [33]. A number of strategies have been proposed for cytokinin manipulation including the regulated expression of *IPT* or *CKX* [3,6,31].

Inhibition of CKX has been suggested as a strategy for enhancing endogenous cytokinin to enhance growth [34] and yield [31,35]. This approach has been successful in enhancing yield components with *ckx* double mutants in *Arabidopsis thaliana* (Arabidopsis) [36] and oil seed rape [37], *OsCKX2* loss-of-function mutants with rice [38], and RNAi gene silencing of *HvCKX1* in barley [39,40], and *TaCKX1* in wheat [41]. Notably, experiments with RNA-guided Cas9 *ckx* mutant lines in barley have shown that single *CKX* gene knockouts might be insufficient for enhancing yield [42]. However, genetically modified plants are not acceptable in several jurisdictions, so alternative methods continue to be sought [43].

CKX-inhibiting compounds, CPPU and TDZ, have long been known to have an effect on the growth of various plant species, with TDZ being an effective cotton defoliant [44,45], and CPPU being able to alter flower morphology [46] and enhance the size of fruits including kiwifruit [47], apple [48] and grape [49]. With the development of CKX-inhibiting compounds [19,35,50], this strategy appears promising.

**Figure 1:**
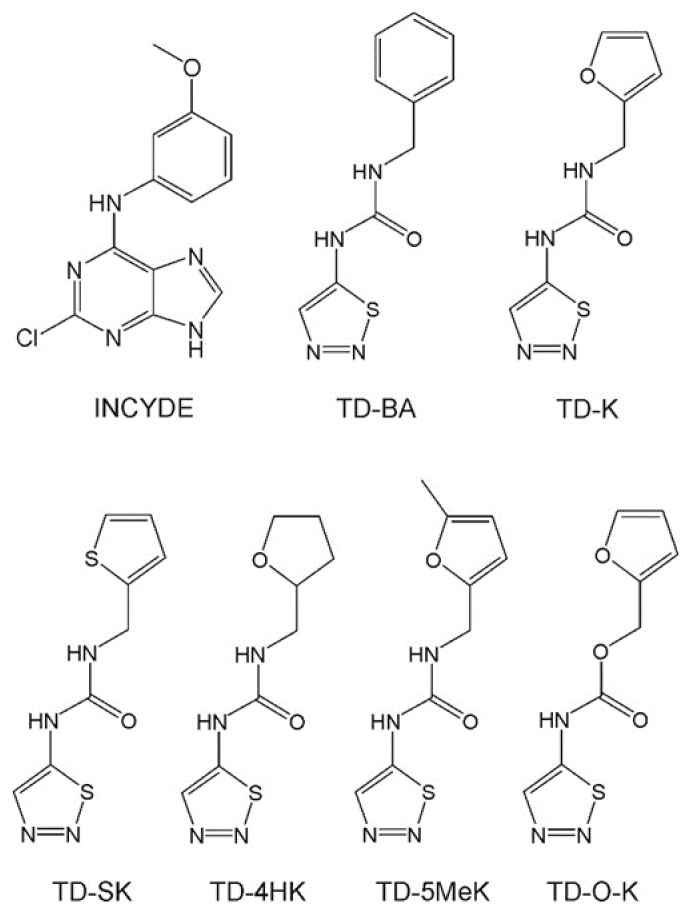
Structures of prepared compounds.

One candidate for increasing the levels of active cytokinins is the substituted 6-anilinopurine derivative, INCYDE (2-chloro-6-(3-methoxyphenyl)aminopurine, Figure 1), a plant growth regulator (PGR) reported to be a potent CKX inhibitor. In contrast to CPPU and TDZ, INCYDE binds only weakly to cytokinin receptors [19], which appears to be its advantage over classical cytokinins [35]. Previous experiments with INCYDE have shown it to increase flower production and enhance antioxidant enzymes in tomato [51]; it helped to alleviate the stress effects from *Verticillium longisporum* [52], salt stress in tomato [51], and cadmium stress in *Rumex crispus* and *Bulbine natalensis* seedlings [34]. This is in agreement with findings provided by Berková et al. [53], who recently showed that INCYDE attenuated abscisic acid signaling and repressed at least 21 additional stress-responsive proteins in Arabidopsis. In *Eucomis autumnalis* subspecies *autumnalis*, INCYDE favored the regeneration of shoots and, relative to PI-55 (a cytokinin antagonist), resulted in the accumulation of higher concentrations of endogenous cytokinin [54]. In hemp (*Cannabis sativa* L.) explants grown on BA, higher concentrations of nucleotide, free base, riboside and storage forms of cytokinins were observed when additionally supplied with INCYDE [55]. In “Williams” banana plantlets, INCYDE, but again in combination with BA, enhanced cytokinin accumulation in aerial parts of the plantlets [56]. It has been reported that Arabidopsis protoplasts treated with INCYDE accumulated both active (*t*Z) and inactivated (iP9G and DHZ9G) cytokinins [57].

However, recent research has also shown contradictory effects of INCYDE. INCYDE decreased the content of cytokinins in non-mycorrhizal control pea plants, particularly the nucleotide forms [58], while in Arabidopsis subjected to heat stress, F-INCYDE (a fluorinated analog of INCYDE, 2-fluoro-6-(3-methoxyphenyl)aminopurine [19]) exacerbated negative stress effects and delayed the stress response of non-acclimated plants [59]. A decrease in cytokinin nucleotide forms was also detected. While there are relatively few examples of direct measurements of cytokinin following treatment of tissues/plants with INCYDE alone, the decreases in nucleotide forms, and increases in storage and/or inactivated forms could be interpreted as the result of homeostatic feedback responses activated as a consequence of elevated cytokinin levels.

Another PGR derived from TDZ, TD-K (1-(furan-2-ylmethyl)-3-(1,2,3-thiadiazol-5-yl)urea, Figure 1), was shown to have practical utilization in agriculture. This compound enhanced drought stress tolerance of winter wheat and, moreover, spray application of TD-K increased seed yield of spring barley and winter oilseed rape in field conditions [60]. In this study we present how TD-K was identified as a potent cytokinin with exceptionally strong anti-senescence activity.

Since both INCYDE and TD-K are compounds which may have practical utilization in agriculture and biotechnology, we were interested in their effects on the expression of genes involved in cytokinin biosynthesis (*IPT*s) and degradation (*CKX*s). We used the model plant Rapid Cycling *Brassica rapa* (RCBr) (Wisconsin Fast Plants^®^) as an alternative to the more commonly used Arabidopsis because it is closely related to several agriculturally important species including turnip (*B. rapa*), rapeseed (*B. napus*), forage brassicas (*Brassica* spp.), cauliflower, broccoli and cabbage (*B. oleracea* cultivars). It has a short life cycle of 40–50 days and is of a manageable size that allows it to be easily harvested [61].

## 2. Results

### 2.1. Design and Chemistry of Compounds

Previously, compounds which are a combination of (1,2,3-thiadiazol-5-yl)urea and isoprenoid chains of cytokinins (compounds TD-iP, TD-*t*Z and TD-DHZ) were shown to exhibit increased anti-senescence activity compared to their related free base [62]. In this work we focused on aromatic side chains, and synthesized compounds TD-BA and TD-K (Figure 1), which combine (1,2,3-thiadiazol-5-yl)urea with the side chains of cytokinins *N*^6^-benzyladenine (BA) and kinetin (K), respectively. Both compounds had been prepared previously, but their anti-senescence activities were not examined [63,64], nor their ^1^H NMR spectra (provided in Supplementary Data S1). Compounds TD-SK, TD-4HK, TD-5MeK and TD-O-K (Figure 1) are derivatives of TD-K and served for investigation of structure-activity relationships in the wheat leaf senescence assay.

### 2.2. Activity in Cytokinin Bioassays

**Figure 2:**
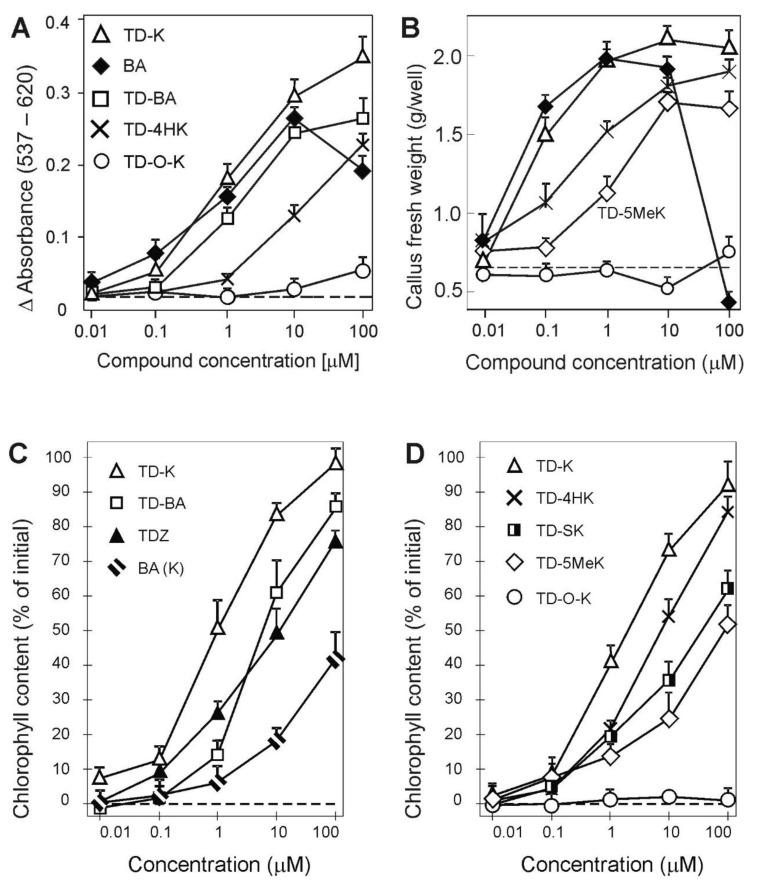
Evaluation of biological activities of the synthesized compounds in classical cytokinin bioassays, compared with the activity of *N*^6^-benzyladenine (BA). Activity of thidiazuron (TDZ) is not shown here to maintain clarity of the graphs, but the activity of TDZ in these bioassays is shown by Nisler et al. [62]. Shown here is the (**A**) effect on dark betacyanin synthesis in *Amaranthus caudatus* L. cotyledon-hypocotyl explants, and the (**B**) effect on growth of cytokinin-dependent tobacco callus. In (**A**) and (**B**), the legend applies to both graphs. In (**C**) and (**D**) a wheat leaf senescence assay was performed in the dark (5 days). The 100% value represents chlorophyll content in fresh control leaves. Dashed lines indicate values obtained for the control treatment (DMSO control) with no added compound. In (**C**) BA (K) indicates that *N*^6^-benzyladenine (BA) and kinetin (K) showed essentially similar activity. Error bars in all graphs show standard deviation of the mean for five replicate determinations. All bioassays were repeated at least twice and the graphs presented are representative examples.

We used three cytokinin bioassays to investigate the complex cytokinin-related properties of the synthesized urea derivatives. The activity of the compounds was compared with that of BA. In the *Amaranthus* assay [65], TD-K showed strong cytokinin activity, which was slightly greater than the activity of BA (Figure 2A). TD-SK exhibited similar activity to TD-K (not shown on the graph). Compound TD-BA showed comparable activity to BA. Activities of TD-4HK and TD-5MeK (not shown in the graph) were almost identical but lower than that of BA. Compound TD-O-K was inactive in this assay.

In the tobacco callus assay, TD-K again showed the highest cytokinin activity and, interestingly, without a cytotoxic effect at 100 μM concentration (Figure 2B). Compound TD-BA (not shown in the graph) had slightly less activity than TD-K. Compounds TD-4HK and TD-5MeK showed less activity than BA. TD-O-K was not active in this assay.

The most interesting results were obtained in the wheat leaf senescence assay, where all but TD-O-K exhibited greater activity than BA or K (Figure 2C,D). The strongest anti-senescence activity was shown by TD-K (IC_50_ = 1.5 µM), followed by TD-BA (IC_50_ = 6.2 µM), TD-4HK (IC_50_ = 7.0 µM), TDZ (IC_50_ = 14 µM), TD-SK (IC_50_ = 30 µM) and TD-5MeK (IC_50_ = 90 µM). Both BA and K had the same activity in this assay with IC_50_ values that exceeded 100 µM. Compound TD-O-K did not exhibit cytokinin-like activity in this assay or in the previous two bioassays.

### 2.3. Activation of Arabidopsis Receptors AHK3 and CRE1/AHK4 and the Cytokinin Primary Response Gene *ARR5*

To shed more light on the cytokinin properties of the studied compounds, they were tested for activation of Arabidopsis cytokinin receptors AHK3 and CRE1/AHK4, and for the ability to activate expression of the cytokinin primary response gene *ARR5*.

**Figure 3:**
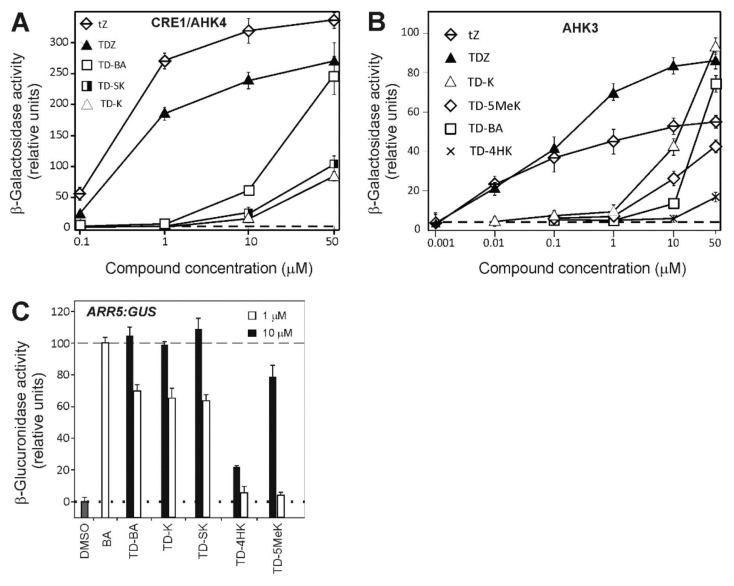
Activation of the cytokinin receptors CRE1/AHK4 (**A**) and AHK3 (**B**) by selected compounds in an *E. coli* receptor activation assay, and quantitative evaluation of β-glucuronidase (GUS) activity in *ARR5:GUS* transgenic Arabidopsis plants (**C**). In (**A**) and (**B**) the activities of the compounds are compared with those of *t*Z and TDZ (used as standards). In (**A**) and (**B**) error bars show standard deviation (*n* = 3). In (**C**) the activity of compounds was compared to the activity of 1 μM BA, which was set as 100% activation (dashed line). DMSO (0.1%) was used as solvent control (dotted line). Error bars show standard deviation of two parallel assays, each consisting of two replicates.

The receptor CRE1/AHK4 was activated by three urea compounds, but only at high concentrations. While 1 μM of either TDZ or *t*Z activated this receptor, 50 μM TD-BA was required to reach a similar activation. Compounds TD-K and TD-SK showed similarly low activation of this receptor, being only slightly active at the highest concentration tested. No other derivative from the series was able to activate the CRE1/AHK4 receptor (Figure 3A).

The receptor AHK3 has a broader ligand specificity and is generally more sensitive than CRE1/AHK4 [18]. Thus, both TDZ and *t*Z activated the AHK3 receptor from as low as 10 nM. The applied compounds activated this receptor, but in a range from 10 to 50 μM. The most active was TD-K, followed by TD-SK (not shown in a graph), TD-5MeK and TD-BA. TD-4HK showed only negligible activity at the highest tested concentration (Figure 3B).

In the *ARR5*:*GUS* reporter gene assay, compounds TD-BA, TD-K and TD-SK exhibited almost identical activity. At 1 μM concentration they reached approximately 70% of the activity exhibited by 1 μM BA. TD-5MeK was approximately 10-fold weaker, requiring to be applied at 10 μM to reach 80% of 1 μM BA activity. The lowest activity was exhibited by TD-4HK (Figure 3C), which was also inactive in the CRE1/AHK4 receptor assay and almost inactive at receptor AHK3. It is important to note that *t*Z and TDZ were previously shown to reach maximal activity in the *ARR5*:*GUS* reporter gene assay at 0.1 μM [62]. From this point of view compounds TD-BA, TD-K and TD-SK are more than 10 times weaker cytokinins than *t*Z or TDZ, while still exhibiting moderate cytokinin properties.

In summary, the assay results presented in Figure 3 are consistent with each other. Compounds TD-BA, TD-K and TD-SK activated both receptors CRE1/AHK4 and AHK3, and also activated *ARR5*. Compound TD-5MeK that activated receptor AHK3 only was also less active in the *ARR5*:*GUS* reporter gene assay than the other three compounds. TD-4HK was the least active compound in these assays, and compound TD-O-K was completely inactive. This fact clearly demonstrates that the urea NH group is necessary for the cytokinin activity of urea-based compounds.

### 2.4. PGR-Treated RCBr

Following four applications of INCYDE, there was a statistically significant increase (*P* ≤ 0.05) in the number of seeds per plant when INCYDE (25 µM) was applied to plants grown in 5 mM KNO_3_ (Table 1). These data are derived from three fully independent experiments. A full data set with KNO_3_ at 0.1, 1 and 10 mM and fertilizer pellets is provided in Table S2. No change in growth or yield was found when TD-K was applied to RCBr (results not shown).

### 2.5. *BrCKX* and *BrIPT* Expression Following True Leaf Appearance

**Figure 4:**
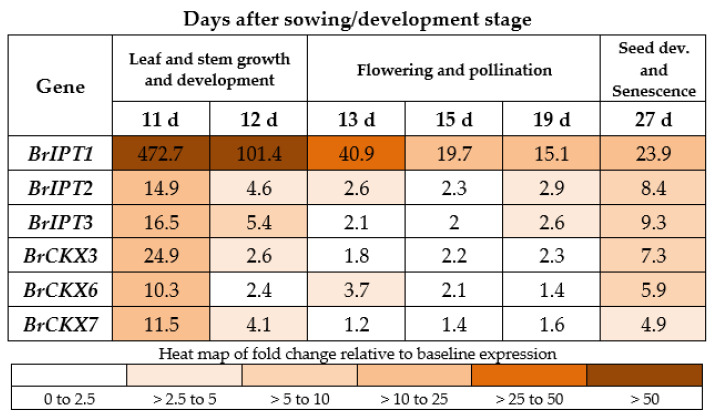
Relative expression of *CKX* and *IPT* gene family members in leaves of control Rapid Cycling *Brassica rapa* plants, represented by fold-change relative to the baseline expression.

Expression of both *BrIPT*s and *BrCKX*s was greatest in leaves at 11 days after sowing, before flowering, with *BrIPT1* showing the strongest expression at each time point measured (Figure 4). Expression levels in leaves declined during flowering and pollination (13 to 19 days after sowing) and then increased later during senescence (27 days after sowing).

### 2.6. *BrCKX* and *BrIPT* Expression Following INCYDE Treatment

**Figure 5:**
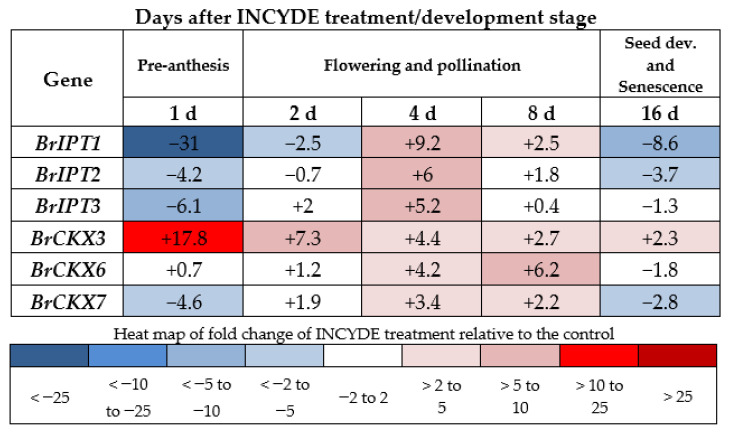
Expression relative to control expression for each of the *CKX* and *IPT* gene family members in leaves of INCYDE-treated Rapid Cycling *Brassica rapa* plants. The positive and negative values represent the fold-change difference between the control and treatment plants.

Within a day following 50 µM INCYDE application, there was a strong upregulation of *BrCKX3* and strong downregulation of *BrIPT1* and to a lesser extent *BrIPT2*, *BrIPT3* and *BrCKX7*, relative to the control group. The marked responses reduced over time, with all gene family members showing positive expression relative to control by 4 d after treatment (Figure 5).

### 2.7. *BrCKX* and *BrIPT* Expression Following TD-K Treatment

**Figure 6:**
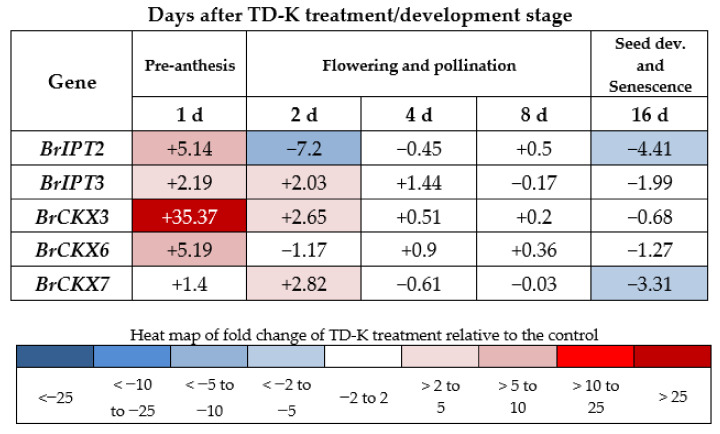
Expression relative to control expression for each of the *CKX* and *IPT* gene family members in leaves of TD-K-treated Rapid Cycling *Brassica rapa* plants. The positive and negative values represent the fold-change difference between the control and treatment plants.

Following TD-K application, there was a strong upregulation of *BrCKX3* and more modest upregulation of other *BrIPT* and *BrCKX* GFMs (Figure 6). This upregulation, relative to the control, was reduced in *BrCKX3* by 2 d after treatment, while in *BrIPT2*, expression was downregulated. By 4 d after treatment, the difference in expression levels between TD-K and the control had disappeared.

## 3. Discussion

TDZ is a molecule that shows a wide range of effects on plants. This is associated with the combination of high cytokinin activity and its CKX-inhibitory activity. Many derivatives of TDZ and their biological properties have been described. The derivatives include compounds with various substituents on the TDZ phenyl ring [50,66], compounds where phenyl is replaced by another heterocycle or (substituted) benzyl ring [50,63,64] and compounds where phenyl is substituted by aliphatic moieties [62]. In this work we present a mini-series of new compounds derived from TD-K and report their biological activity. In this respect, TD-K exhibited the highest cytokinin activity of all the derivatives tested. However, in the *Amaranthus* and tobacco callus bioassays the activity of TD-K is very similar to that of BA. This means that TD-K is, in these two assays, less active than TDZ or *t*Z (data shown in Nisler et al. [62]). This is in agreement with Mok et al. [63] who showed that TD-K was less effective than TDZ and *t*Z in promoting the growth of cytokinin-dependent callus of *Phaseolus lunatus*. TD-K and TD-BA were also shown to be less effective than TDZ in cytokinin-dependent ethylene production systems [64]. These lesser responses are most probably the reason why the biological activity of TD-K and TD-BA was not further investigated in the 1980s.

We synthesized these compounds in order to examine their activity in the wheat leaf senescence assay. Here, TD-K showed the strongest anti-senescence activity in this assay of all compounds (3000+) ever tested in the Laboratory of Growth Regulators. TD-K is by one order of magnitude more effective than TDZ and by two orders of magnitude more effective than BA or K. The other compounds from the series presented here also showed very high anti-senescence activity. Of particular note, is the result achieved with TD-4HK. This compound, possessing a saturated tetrahydrofuranyl ring, showed only negligible cytokinin activity in Arabidopsis but retards chlorophyll degradation in wheat leaves with greater efficacy than TDZ. It demonstrates that even compounds without a second aromatic ring (the 1,2,3-thiadiazolyl ring must be present) can be effective in the wheat leaf senescence assay. Based on this observation, TDZ derivatives with aliphatic substitutions (optionally containing an oxygen atom) instead of a phenyl ring were prepared, and their strong anti-senescence activity was revealed [62]. Such compounds, including TD-4HK, exhibited only weak cytokinin activity when compared to TD-K. For this reason, we selected TD-K for investigation of its effect on expression of GFMs associated with cytokinin homeostasis.

Another plant growth regulator potentially able to supply plants with an enhanced cytokinin signal is INCYDE. When compared to TD-K, INCYDE exhibits weaker cytokinin activity [19] but, on the other hand, by inhibiting CKX it could increase the levels of endogenous cytokinins over a longer period of time. It is, therefore, likely that the mechanisms by which TD-K and INCYDE alter cytokinin signaling in plants are different. For this reason, we were interested in the impact of these compounds on gene expression in whole plants. We initially applied TD-K and INCYDE to determine their effects on growth and yield under controlled conditions. Overall, there was little evidence of changes in growth or yield following application of either TD-K or INCYDE. Yield enhancement (seeds per plant) was not caused by a change in apical dominance and was only evident when INCYDE was applied multiple times before flowering (at growth stages one to five; [67]) and when grown with 5 mM KNO_3_ (Table 1). This result is, in some respects, in contrast to the results described by Nisler et al. [35], who showed that a single application of the CKX inhibitor 3TFM-2HE (based on diphenylurea) was able to increase seed yield in Arabidopsis. This inhibitor, however, exhibited much higher inhibitory activity than F-INCYDE with three out of four CKX tested. It is, however, possible that the lack of effect generally was due to the plants growing under optimal conditions, with little capacity for further enhancement of growth.

We noted that, recently, there are contrasting reports on the efficacy of INCYDE in response to biotic and abiotic stressors [34,51,59], as well as reports showing both enhanced [54,55] and decreased content of cytokinins [58].

Targeting cytokinin homeostasis through the application of exogenous compounds leads to a range of effects that are frequently inconsistent [68], which supports suggestions that cytokinin homeostasis is complex and tightly regulated. It is well known that targeting CKX with CKX-inhibiting PGRs has an effect on cytokinin homeostasis and/or cytokinin-associated processes [44,45,46,47,48,49], including INCYDE [34,51,52,54,56] and through targeting CKX in mutants and/or transgenic plants [36,37,38,39,41,42,69].

We undertook the expression studies to determine whether INCYDE and TD-K disrupted cytokinin homeostasis by affecting the expression of GFMs associated with cytokinin biosynthesis and degradation, and whether this could explain the lack of effect of INCYDE and TD-K on our RCBr plants. Initially we showed that the differences in the levels of expression between young leaves, expanded leaves and leaves undergoing senescence in the control generally aligned with previous research with RCBr [61]. The strongest expression of *BrCKX* GFMs occurred before flowering (Figure 3A). As reduced expression of *CKX* GFMs in the reproductive shoot apical meristem was associated with yield enhancement in Arabidopsis [36], it was appropriate to target INCYDE before flowering in both the yield and expression experiments.

The strong, upregulation of *BrCKX3* within a day following INCYDE treatment was unexpected but indicates a feedback response that could normalize cytokinin levels. Moreover, *BrCKX3* remained upregulated during the whole course of the experiment (16 days), suggesting that following INCYDE inhibition of CKX, an elevation of cytokinin occurred during the lifespan of RCBr. An upregulation of *CKX* expression and/or activity has been observed elsewhere following increases in cytokinin levels [70,71,72,73,74], with evidence also of expression of *CKX* GFMs correlating with endogenous cytokinin levels in developing tissues [38,75,76]. Nisler et al. [35] also reported fast (within hours) upregulation of CKX GFMs after application of their CKX inhibitor and mild upregulation of ARR GFMs 24 h after treatment, indicating that application of the CKX inhibitor increased the content of active cytokinins in developing seedlings. The downregulation of *BrIPT* GFMs, particularly *BrIPT1*, one day post-treatment also forms a part of this feedback response. This aligns with other research which has observed a downregulation of *IPT*s following an enhancement of cytokinin through direct application with BA [21,74]. Thus, our data are in accordance with the discussed literature and suggest that INCYDE increased the level of active cytokinin forms in RCBr.

To compare the effect of INCYDE, TD-K was applied at a similar growth stage. TD-K application also led to a strong upregulation of *BrCKX3* compared to the control, but it lasted only one day, suggesting that TD-K provided RCBr with a short cytokinin signal. It is not known if or how TD-K could be inactivated in plants, but the results show that the expression profile of the studied genes was normalized within two days after TD-K application. This is in contrast to the longer-term effect of INCYDE application. Previous studies have predicted the location of *Brassica rapa* ssp. Pekinensis BrCKX3 protein in chloroplasts and secretory pathways [74], while in *Brassica napus* L. BnCKX3 was predicted to be in the vacuole [77], and confirmed to be vacuole-based in Arabidopsis [4]. However, the over-production of vacuole-located AtCKXs (AtCKX1 and AtCKX3) is known to cause severe traits associated with cytokinin deficiency in Arabidopsis [4], highlighting the potential importance of *BrCKX3* in regulating cytokinin homeostasis and cytokinin sink dynamics at an intracellular level. Interestingly, expression of *CKX3* was the most strongly upregulated *CKX* GFM in Arabidopsis *N*-glucosyl transferase mutants [78].

## 4. Conclusions

Both compounds INCYDE (CKX inhibitor) and TD-K (cytokinin) impact expression of *IPT* and *CKX* GFMs in RCBr, albeit with different intensity and over a different time span. The effect of INCYDE on BrCKX3 was gentler and lasted for a longer period of time than the effect of TD-K. However, of all CKXs analyzed, *BrCKX3* showed the greatest change in expression as a response to INCYDE and TD-K treatment. Thus, in respect to the discussed literature, we suggest that *BrCKX3* may well play a key role in responding to cytokinin homeostasis disruption in RCBr, with a downstream impact on other cytokinin-associated processes.

## 5. Materials and Methods

### 5.1. Chemical Synthesis

The compound 1,2,3-thiadiazol-5-amine (TCI Europe) was the starting material for the synthesis of 5-isocyanato-1,2,3-thiadiazole, which was prepared as described by Nisler et al. [50]. All compounds presented here were prepared by mixing 5-isocyanato-1,2,3-thiadiazole and the corresponding commercially available amine (or furan-2-ylmethanol in case of TD-O-K) under mild heat (30 to 60 °C) in tetrahydrofuran. In addition to this, TD-O-K was synthesized in the presence of a catalytic amount of triethylamine [79]. The conversion process was monitored by thin layer chromatography. The reaction lasted from 1 h to 3 h. After the reaction, the solvent was evaporated, and the resulting solid residue was then purified by column chromatography using silica gel (Merk) and CHCl_3_:MeOH (9:1) mobile phase. The yields were about 60%.

All compounds were prepared according to common protocols for the synthesis of bis-substituted urea derivatives [79].

### 5.2. Cytokinin Bioassays, Cytokinin Receptor Activation Assay and Arabidopsis ARR5:GUS Reporter Gene Assay

Tobacco (*Nicotiana tabacum* L.) callus, *Amaranthus* (*Amaranthus caudatus* L.) and wheat (*Triticum aestivum* L.) leaf senescence assays were performed as described by Holub et al. [80] with minor modification in the tobacco callus bioassay [62]. The *Amaranthus* assay determines the quantity of cytokinin-induced betacyanin synthesis in *Amaranthus*. The cytokinin receptor activation assays and the Arabidopsis *ARR5:GUS* reporter gene assay were done exactly as described by Nisler et al. [62].

### 5.3. Plant Material

Rapid Cycling *B. rapa* seeds (Wisconsin Fast Plants^®^, Madison, WI, USA) were sown in small pots containing general potting mix and placed into treatment trays. Trays were continually provided with 0.1, 1, 5 and 10 mM KNO_3_ dissolved in water, except in the case of plants provided with Tui Novatec Premium fertilizer, which were provided with water. Plants were grown in growth rooms at 22°C with 24 h of continuous lighting (photosynthetic photon flux density (PPFD) 200–300 µmol m^−2^s^−1^). Plants were hand pollinated from day 13 after sowing. INCYDE was dissolved in minimal DMSO prior to dilution with water for application. For surfactants, either 0.1% Tween 20 or Silwet L-77 was added to the solutions. Whole plants were sprayed until runoff. INCYDE was applied four times at 8, 9, 10 and 12 days after sowing following true leaf appearance. TD-K was applied once before flowering at 10 days after sowing.

Rapid Cycling *B. rapa* plants used for RT-qPCR analyses were provided with 1 mM KNO_3_, and sprayed once at 11 days from sowing, developmentally between the stages of true leaf appearance and before flowering, with 50 µM INCYDE or 50 µM TD-K mixed with a 0.1% Silwet L-77. Given the small size of upper leaves early in development, only the lower leaves (which were ~20 mm in size) were harvested. Plants were first sampled at 11 days after sowing, and 2 h prior to treatment, and subsequently at 1, 2, 4, 8 and 16 days after treatment by harvesting true leaves, flash freezing in liquid nitrogen, grinding to a powder and storing at −80°C.

### 5.4. Gene Expression

#### 5.4.1. RNA Isolation

To extract RNA from the INCYDE and TD-K treated leaves, an RNeasy Plant Mini Kit (Qiagen, Hilden, Germany) was used following the manufacturer’s protocol using the RLT buffer. To prevent contamination, DNA was removed using RNase-Free DNase (Qiagen). Extracted RNA was stored at −20°C. The purity and concentration of the total RNA were determined using a NanoDrop^®^ ND-1000 spectrophotometer (Thermo Fisher Scientific, Waltham, MA, USA). Samples with a value > 1.8 at 260/280 nm and 260/230 nm ratios were selected. The integrity of the RNA was checked using electrophoresis on a 1% (*w*/*v*) agarose gel.

#### 5.4.2. cDNA Synthesis

Up to 1 µg of RNA was converted into cDNA by producing a 10 µL primer annealing mix using 25x RNA secure™ (Thermo Fisher Scientific, Waltham, MA, USA), 100 pmol of Random pd(N)_6_ (Sigma-Aldrich, St Louis, MO, USA), 50 pmol Oligo(dT)_18_ primers ((Meridian Bioscience Inc., Cincinnati, OH, USA) and DEPC-treated water. This was mixed with RT buffer (Sigma-Aldrich), 20 mM dNTP (Thermo Fisher Scientific), DTT (Sigma-Aldrich) and Expand™ Reverse Transcriptase (Sigma-Aldrich) and made up to 20 µL with DEPC-treated water. Samples were then incubated with a Mastercycler^®^ pro (Eppendorf) at room temperature (10 min), 42 °C for 60 min and at 70 °C for 15 min, before a final 10-fold dilution with UltraPure DNase/RNase-Free Distilled Water (Thermo Fisher Scientific) and storage at −20 °C.

#### 5.4.3. Reverse Transcriptase Quantitative PCR

RT-qPCR essentially followed Song et al. [76] using a SYBR green home-made mix or a KAPA SYBR^®^ FAST qPCR Master Mix (2x) (Sigma-Aldrich, St Louis, MO, USA). RT-qPCR reactions were carried out used a real-time PCR cycler Rotor-Gene Q (Qiagen, Hilde, Germany), with 40 cycles at 95°C for 10 s, between 52°C to 64°C for 15 s and 72°C for 15 s, and analyzed with Rotor-Gene Series Software 2.3.1 (Qiagen, Hilden, Germany). The PCR products were sent to Macrogen for sequencing and aligned using National Centre for Biotechnology Information (NCBI) databases with target genes.

#### 5.4.4. Expression Analyses

For internal controls, two reference genes were used: glyceraldehyde-3-phosphate dehydrogenase (*BrGAPDH*) and elongation factor (*BrELF1*) [61,81]. These were used to generate a correction factor and to calculate the expression of target genes using the approach described in Song et al. [76] and based on Pfaffl [82]. Two biological replicates were used with three technical replicates for each biological replicate. A subsample of ten plants made up each biological replicate. Two types of heat maps were generated from expression data. The first set included the natural expression change occurring in RCBr over time. This involved using the control samples only and calculating the expression over time relative to the baseline expression as described in Song et al. [76]. For the second set of heat maps, the expression change of treatment samples was compared with control and presented as up or down regulation in the treatment compared to the control.

### 5.5. Experimental Design and Statistical Analyses

Growth room experiments were arranged in a randomized complete block design blocked for treatments. Each treatment was replicated three times. Each replicate involved a subsample of 30 plants for the yield/growth measurements. Overall means for each treatment were generated using replicate means and reported with the standard error.

For yield and dry weight, a two-sided ANOVA analysis was carried out. For the INCYDE treatment experiment, count data were analyzed with a two-sided Poisson regression (log-link, CI: 95%). Interaction plots were used to determine if there was an interaction effect between different factors. ANOVA assumptions were met through examination of a Q-Q plot of standardized residuals, and homoscedasticity ensured through plotting standardized residuals against predicted values.

## Figures and Tables

**Table 1:** The effect of four applications of 25 µM INCYDE on shoot dry weight (DW), branch number, silique number, silique length, mass, seeds per silique, seeds per plant and seed mass in Rapid Cycling *Brassica rapa* supplied with 5 mM KNO_3_ solution.

Trait	Control	INCYDE
Shoot DW (mg)	183.9 ± 96.9 ^a^	186.2 ± 91.9 ^a^
Branch number	1.6 ± 0.03 ^a^	1.8 ± 0.09 ^a^
Silique number	5.2 ± 1.4 ^a^	7.0 ± 2.3 ^a^
Silique length (mm)	37.2 ± 1.6 ^a^	39.7 ± 0.8 ^a^
Silique mass (mg)	50.8 ± 2.1 ^a^	46.6 ± 1.2 ^a^
Seeds per silique	11.9 ± 1.2 ^a^	12.3 ± 2.6 ^a^
Seeds per plant	54.3 ± 7.0 ^b^	74.5 ± 4.8 ^a^
Seed mass (mg)	1.6 ± 0.8 ^a^	2.3 ± 0.4 ^a^

Means with different letters indicate a statistically significant difference for the treatment compared to the control with Poisson regression (log–link, CI: 95%). Data are presented as the means ± standard error (*n* = 3).

## Data Availability

All data are contained within the article or supplementary material.

## Supplementary Materials

The following are available online at https://www.mdpi.com/2223-7747/10/1/39/s1, Supplementary S1: NMR spectra of five novel thidiazuron derivatives, Table S2: The effect of four applications of 25 µM INCYDE on shoot DW, silique number, silique length, mass, seeds per silique, seeds per plant and seed mass in rapid-cycling *Brassica rapa* supplied with 0.1, 1, 10 mM KNO_3_ solution or fertilizer pellets.
