# Supplementary material for: Targeting Cytokinin Homeostasis in Rapid Cycling Brassica rapa with Plant Growth Regulators INCYDE and TD-K
Source: Plants (Basel). 2020 Dec 25;10(1):39. doi: 10.3390/plants10010039 (PMC7824111; doi:10.3390/plants10010039)
Supplement: Supplementary file 1 [file plants-10-00039-s001.pdf]

S1. NMR spectra for the thidiazuron derivatives.

1. **1. 1-Benzyl-3-(1,2,3-thiadiazol-5-yl)urea (TD-BA):** ^{1}H NMR (δ, ppm, DMSO-d_6): 4.33 (2H, d, J=6.0 Hz, CH_2) 7.21 (1H, tt, J_1=7.2 Hz, J_2=1.4 Hz, ArH-benzyl), 7.24-7.32 (4H, m, ArH-benzyl), 7.57 (1H, t(br) J=6.0 Hz, NH), 8.46 (1H, s, ArH-thiadiazol), 10.84 (1H, s(br), NH); m. p. 179 - 183 °C.
2. **2. 1-(Furan-2-ylmethyl)-3-(1,2,3-thiadiazol-5-yl)urea (TD-K):** ^{1}H NMR (δ, ppm, DMSO-d_6): 4.32 (2H, d, J=5.5 Hz, CH_2), 6.24 (1H, d, J=4.0 Hz, ArH-furfuryl), 6.36 (1H, t, J=2.0 Hz, ArH-furfuryl), 7.52 (1H, t, J=5.5, NH), 7.56 (1H, d, J=4.0 Hz, ArH-furfuryl), 8.47 (1H, s, ArH-thiadiazol), 10.76 (1H, s(br), NH); m.p. 195-199°C.
3. **3. 1-(1,2,3-Thiadiazol-5-yl)-3-(thiophen-2-ylmethyl)urea (TD-SK):** ^{1}H NMR (DMSO-d_6): 4.48 (2H, d, J=2.5 Hz, CH_2), 6.93 (1H, t, J=4.0 Hz, ArH-thiophene), 6.97 (1H, d, J=1.5 Hz, ArH-thiophene), 7.36 (1H, d, J=3.0 Hz, ArH-thiophene), 7.66 (1H, t, J=4.5, NH), 8.46 (1H, s, ArH-thiadiazol), 10.86 (1H, s(br), NH); m.p. 197-201°C.
4. **4. 1-((Tetrahydrofuran-2-yl)methyl)-3-(1,2,3-thiadiazol-5-yl)urea (TD-4HK):** ^{1}H NMR (DMSO-d_6): 1.78 (2H, octet, J=6.9 Hz, CH_2), 1.84 (1H, sextet, J=4.6 Hz, CH), 3.13 (1H, td, J_d=13.2 Hz, J_t=5.7 Hz, CH_2), 3.22 (1H, td, J_d=13.2 Hz, J_t=5.7 Hz, CH_2), 3.34 (1H, q, J=7.45, CH_2), 3.59 (1H, q, J=7.45 Hz, CH_2), 3.73 (1H, q, J=8.0 Hz, CH_2), 3.85 (1H, dq, J_d=6.9 Hz, J_q=5.5 Hz, CH_2), 7.02 (1H, s(br), NH), 8.47 (1H, s, ArH-thiadiazol), 10.62 (1H, s, NH); m.p. 173 - 177°C.
5. **5. 1-((5-Methylfuran-2-yl)methyl)-3-(1,2,3-thiadiazol-5-yl)urea (TD-5MeK):** ^{1}H NMR (DMSO-d_6): 2.18 (3H, s, CH_3), 4.25 (2H, d, J=5.7 Hz, CH_2), 5.95 (1H, q, J=1.2 Hz, ArH-furfuryl), 6.10 (1H, d, J=2.9 Hz, ArH-furfuryl), 7.50 (1H, t, J=4.5 Hz, NH), 8.46 (1H, s, ArH-thiadiazol), 10.75 (1H, s(br), NH); m. p. 147 - 151°C.
6. **6. Furan-2-ylmethyl (1,2,3-thiadiazol-5-yl)carbamate (TD-O-K):** ^{1}H NMR ($CDCl_3$-d_1): 5.26 (2H, s, CH_2), 6.38 (1H, t, J=3.4 Hz, ArH-furfuryl), 6.51 (1H, d, J=3.4 Hz, ArH-furfuryl), 7.45 (1H, d, J=1.2 Hz, ArH-furfuryl), 8.25 (1H, s(br), NH), 8.44 (1H, s, ArH-thiadiazol); m.p. not determined.

Table S2. The effect of four applications of 25 µM INCYDE on shoot DW, silique number, silique length, mass, seeds per silique, seeds per plant and seed mass in rapid-cycling *Brassica rapa* supplied with 0.1, 1, 1 mM KNO_3 solution or fertiliser pellets.

	**KNO_3 0.1 mM**	
**Trait**	**Control**	**INCYDE**
Shoot DW (mg)	8.8 ± 1.1	15.5 ± 5.5
Silique number	0.6 ± 0.1	0.6 ± 0.1
Silique length (mm)	25.3 ± 0.3	26.2 ± 1.6
Silique mass (mg)	20.0 ± 1.6	22.5 ± 2.3
Seeds per silique	6.0 ± 0.5	5.4 ± 0.6
Seeds per plant	3.4 ± 0.6	3.3 ± 1.0
Seed mass (mg)	2.1 ± 0.2	2.2 ± 0.01
	**KNO_3 1 mM**	
**Trait**	**Control**	**INCYDE**
Shoot DW (mg)	26.1 ± 10.5	25.1 ± 11.8
Silique number	2.2 ± 0.5	1.9 ± 0.7
Silique length (mm)	34.0 ± 1.8	32.5 ± 0.6
Silique mass (mg)	37.9 ± 1.1	35.4 ± 3.4
Seeds per silique	9.3 ± 0.6	11.2 ± 0.1
Seeds per plant	19.1 ± 2.7	21.3 ± 7.6
Seed mass (mg)	2.3 ± 0.1	2.0 ± 0.2
	**KNO_3 10 mM**	
**Trait**	**Control**	**INCYDE**
Shoot DW (mg)	222.2 ± 56.5	247.5 ± 50.2
Silique number	6.4 ± 0.8	5.7 ± 0.7
Silique length (mm)	37.4 ± 0.8	35.7 ± 1.8
Silique mass (mg)	38.8 ± 3.5	42.7 ± 4.4
Seeds per silique	12.9 ± 1.5	12.4 ± 2.7
Seeds per plant	78.3 ± 2.2	73.1 ± 1.0
Seed mass (mg)	2.0 ± 0.3	2.1 ± 0.3
	**Fertiliser pellets**	
**Trait**	**Control**	**INCYDE**
Shoot DW (mg)	141.3 ± 101.2	157.0 ± 117.7
Silique number	5.1 ± 1.6	5.5 ± 2.2
Silique length (mm)	35.6 ± 4.2	33.6 ± 2.2
Silique mass (mg)	35.0 ± 4.2	32.4 ± 1.6
Seeds per silique	12.8 ± 1.6	11.9 ± 1.3
Seeds per plant	59.1 ± 9.5	58.1 ± 18.2
Seed mass (mg)	1.7 ± 0.3	1.7 ± 0.1

Data is presented as the means ± standard error (*n* = 3).
